# Novel Harmonic Regularization Approach for Variable Selection in Cox's Proportional Hazards Model

**DOI:** 10.1155/2014/857398

**Published:** 2014-11-24

**Authors:** Ge-Jin Chu, Yong Liang, Jia-Xuan Wang

**Affiliations:** University Hospital, State Key Laboratory of Quality Research in Chinese Medicines, Faculty of Information Technology, Macau University of Science and Technology, Macau

## Abstract

Variable selection is an important issue in regression and a number of variable selection methods have been proposed involving nonconvex penalty functions. In this paper, we investigate a novel harmonic regularization method, which can approximate nonconvex *L*q  (1/2 < *q* < 1) regularizations, to select key risk factors in the Cox's proportional hazards model using microarray gene expression data. The harmonic regularization method can be efficiently solved using our proposed direct path seeking approach, which can produce solutions that closely approximate those for the convex loss function and the nonconvex regularization. Simulation results based on the artificial datasets and four real microarray gene expression datasets, such as real diffuse large B-cell lymphoma (DCBCL), the lung cancer, and the AML datasets, show that the harmonic regularization method can be more accurate for variable selection than existing Lasso series methods.

## 1. Introduction

One of the most important objectives for survival analysis is to select a small number of key risk factors from many potential predictors [1]. Commonly, the Cox proportional hazards model [2, 3] is used to study the relationship between predictor variables and survival time. Suppose a dataset has a sample size of *n* to study the survival time *T* on covariate *x*; we use the data form of (*t*_1_, *δ*_1_, *x*_1_),…, (*t*_*n*_, *δ*_*n*_, *x*_*n*_) to represent the individual's sample, where the survival time *t*_*i*_ being complete if *δ*_*i*_ = 1 and right censored if *δ*_*i*_ = 0. As in regression, *x*_*i*_ = (*x*_*i*1_, *x*_*i*2_,…, *x*_*ip*_) is a potential prediction vector.

By Cox's proportional hazards model, the hazard function is given as

(1) $$\begin{array}{c} h\left(t\;|\;\beta \right)=h_{0}\left(t\right)\exp\left(x^{T}\beta \right), \end{array}$$

where the baseline hazard function *h*_0_(*t*) is unspecified and *β* = (*β*_1_, *β*_2_,…, *β*_*p*_)^*T*^ is the regression coefficient vector of *p* variables. Cox's partial log-likelihood is expressed as

(2) $$\begin{array}{c} l\left(\beta \right)=\overset{n}{\underset{i=1}{\sum }}\delta_{i}\left\{x_{i}^{T}\beta -\log\left(\sum_{j\in R_{i}}\exp\left(x_{i}^{T}\beta \right)\right)\right\}, \end{array}$$

where *R*_*i*_ denotes the set of indices of the survival individuals at time *t*_*i*_.

In practice, only a small number of the predictor variables actually affect the hazard rate. The goal of variable selection in Cox's proportional hazards model is to select the key risk factors. Recently a series of penalized partial likelihood methods, such as the *L*_1_ [4–7], *Lq* (0 < *q* < 1) [8] and *L*_1/2_ [9, 10] penalized methods were proposed for Cox's proportional hazards model. These penalized partial likelihood methods find important risk factors by shrinking some regression coefficients to zero.

The standard penalized methods cannot directly be applied to the nonlinear Cox model to obtain parameter estimates. Therefore, Tibshirani [11] proposed an iterative procedure to transform the Cox's partial log-likelihood function (2) to linear regression problem. Let *x* = (*x*_*i*1_,…, *x*_*ip*_)^*T*^, *i* = 1,…, *n*, and denote the *p* × *n* predictor variable matrix, *η* = *x* ^*T*^ *β*, *μ* = −(∂*l*/∂*η*), *A* = −(∂^2^ *l*/∂*η*∂*η* ^*T*^), and *z* = *η* + *A* ^−^ *μ*, where *A* ^−^ is a generalized inverse of *A*. Since the general quadratic programming cannot be directly solved to the cases with *p* ≫ *n*, Gui and Li [12] applied the Choleski decomposition to obtain *C* = *A* ^1/2^ such that *C* ^*T*^ *C* = *A*, $\hat{y}=Cz$, and $\hat{x}=Cx$. By the Taylor expansion, the partial log-likelihood *l*(*β*) is approximated by the quadratic form:

(3) $$\begin{array}{c} {\left(\hat{y}-{\hat{x}}^{T}\beta \right)}^{T}\left(\hat{y}-{\hat{x}}^{T}\beta \right). \end{array}$$

Thus, the regularization methods can directly solve the penalized regression problem:

(4) $$\begin{array}{c} \hat{\beta }=\underset{\beta }{\arg\;\min}\left({\left\|\hat{y}-{\hat{x}}^{T}\beta \right\|}^{2}+\lambda \overset{p}{\underset{j=1}{\sum }}P\left(\beta_{j}\right)\right), \end{array}$$

where *λ* is the tuning parameter.

Tibshirani [5] proposed the Lasso (least absolute shrinkage and selection operator) method, which has *L*_1_ penalty *P*(*β*_*j*_) = |*β*_*j*_|, which shrinks small coefficients to zero and hence results in a sparse representation of the solution. Fan and Li [4] proposed the smoothly clipped absolute deviation (SCAD) penalty, which avoids excessive penalties on large coefficients and enjoys the oracle properties. Zhang [7] proposed the minimax concave plus (MCP) method, which is a continuous and nearly unbiased approach in high-dimensional linear regression. Zhang and Lu [6] suggested an adaptive Lasso method with an adaptively *L*_1_ penalty estimate the parameters, which uses the penalty *P*(*β*_*j*_) = |*β*_*j*_ | /|*β*_*j*_′|, where the weights 1/|*β*_*j*_′| are chosen adaptively by the data. Zou and Hastie [13] proposed an elastic net method that combines the *L*_1_ and *L*_2_ (*P*(*β*_*j*_) = |*β*_*j*_|^2^) penalties.

The above mentioned series of regularized regression methods were based on the *L*_1_ penalty. Recently, several works on learning sparse models have stressed the need of other penalties for achieving better sparsity profile. For instance, Rosset and Zhu [14] suggested the use of a *Lq* penalty, which simply consists in replacing the *L*_1_ norm with nonconvex *Lq* norm (0 < *q* < 1). Zhang [15] presented a multistage convex relaxation scheme, which can be relaxed to a smoothed *Lq* regularization. Mazumder et al. [16] pursued a coordinate-descent approach with nonconvex penalties (SparseNet) and study its convergence properties. Xu et al. [9, 10] further explored the properties of the *Lq* (0 < *q* < 1) penalty and revealed the extreme importance and special role of the *L*_1/2_ regularization. In our previous work [17, 18], we developed several fast algorithms using the *L*_1/2_ penalty to solve the logistic regression model and the Cox model. Our computational results showed that *L*_1/2_ regularization outperforms some *L*_1_ regularization methods. In this paper, we propose a novel harmonic regularization method which approximates to the *Lq* (1/2 < *q* < 1) penalties. We also investigate the fast harmonic regularization algorithm to solve the Cox model for the high dimension low sample size problem (“large *p* small *n* problem”).

The rest of the paper is organized as follows.  Section 2 describes the harmonic regularization method.  Section 3 gives a harmonic regularization algorithm to obtain estimates form Cox model.  Section 4 evaluates our method by simulation studies and application to four real microarray datasets, such as the diffuse large B-cell lymphoma (DLBCL) datasets with the survival times and gene expression data.  Section 5 concludes the paper with some useful remarks.

## 2. Harmonic Regularization

In general, a united framework of the regularization in machine learning has a form:

(5) $$\begin{array}{c} \hat{\beta }\left(\lambda \right)=\underset{\beta }{\arg\;\min}\left[R\left(\beta \right)+\lambda P\left(\beta \right)\right], \end{array}$$

where *R*(*β*) is a loss function, *P*(*β*) is a penalty function, and *λ* is a tuning parameter. Different *λ* here is in correspondence with different penalized constraint to the model, so different solution is to be got, respectively. The penalized constraint is the weakest when *λ* = 0 and becomes stronger as *λ* increases.

Obviously a regularization (5) can be divided by two elements, the loss function *R*(*β*) and the penalty function *P*(*β*). Moreover, different loss function and different penalty will result in different algorithm. For example, when the loss function is hinge loss and the penalty *P*(*β*) = ‖*β*‖^2^, the result is a support vector machine algorithm. Let the loss function be square loss and using *P*(*β*) = ‖*β*‖^*q*^ denote the *Lq* regularization methods, if *q* = 2, it is the ridge regression [19] and can be used to solve the ill-posed problem. If *q* = 0, it is the subsets regression [20], which applies *L*_0_ regularization with the penalty function *P*(*β*) = (1/2)*I*_(|*β*≠0|)_. When *q* = 1, it is the Lasso algorithm [21], which applied *L*_1_ regularization. Lasso and its variations (or the Lasso type algorithms), such as elastic net [13], SCAD [4], MCP [7], adaptive Lasso [6], and stage-wise Lasso [22] are extensively studied and applied in recent years in the fields of statistics and machine learning.

It is well known that *L*_0_ regularization is ideal sparsest for variable selection. Unfortunately, *L*_0_ regularization is a combinatorial optimization problem, which is difficult to be solved. In contrast, *L*_1_ regularization leads to a convex optimization problem and easy to be solved, but it does not yields sufficiently sparse variable selection. Donoho et al. [23, 24] had shown that *L*_0_ regularization is equivalent to *L*_1_ regularization under certain conditions. These imposed conditions therefore characterize those problems for which no matter what *L*_1_ or *L*_0_ regularization is applied, the same sparse solutions will be produced. However, for many practical problems, the sparsity of solutions yielded through *L*_1_ and *L*_0_ regularization is far from being equivalent. Particularly, the solutions found with *L*_1_ regularization is very often less as sparse as the solutions found with *L*_0_ regularization.

In fact, when 0 ≤ *q* ≤ 1, the *Lq* regularization automatically performs variable selection by removing predictors with very small nonzero estimated coefficients. The smaller the *q* is, the sparser the solutions found with *Lq* regularization will be. This leads researchers to study *Lq* regularization with 0 < *q* < 1 because it can find the more sparse solutions than those found with *L*_1_ regularization and easier to be solved than *L*_0_ regularization. For example, Zhang [15] presented a multistage convex relaxation scheme for solving problems with nonconvex objective functions. For learning formulations with sparse regularization, they analyzed the behavior of a specific multistage relaxation scheme.

Nevertheless, the applications to the *Lq* penalty function with 0 < *q* < 1 not often attracts much attention done mainly due to the reason that when 0 < *q* < 1, the penalty function changes from a convex function to a nonconvex one and so the corresponding optimization problem is not easy to solve. Meanwhile, another difficulty in fact is that the differential quotient of the penalty function at origin is +*∞* which results in the invalidation of the ordinarily optimization algorithms.

In this paper, we propose the harmonic regularization which can approximate the *Lq* penalty with 1/2 ≤ *q* < 1, because some research works show that the *L*_1/2_ penalty can be taken as a representative of the *Lq* (0 < *q* < 1) penalty [22]. The harmonic regularization scheme can be expressed as

(6) β^=arg min⁡β1n∑i=1ny^i−x^iβ22aa−1βi+2−aa−12ddddddddid+λ∑i=1p2aa−1βi+2−aa−12−2−aa−1,

where 1 < *a* < 2. When the shrinkage parameter *a* is close to 1, $\sqrt{(2/a\left(a-1\right))|\beta |+{\left(\left(2-a\right)/(a-1)\right)}^{2}}-\left((2-a\right)/\left(a-1\right))\approx |\beta |$, the harmonic regularization approximates to the *L*_1_ regularization. When the parameter *a* is close to 2, $\sqrt{(2/a(a-1))|\beta |+{\left((2-a)/\left(a-1\right)\right)}^{2}}-\left((2-a)/\left(a-1\right)\right)\approx \sqrt{\left|\beta \right|}$ and the harmonic regularization approximates to the *L*_1/2_ regularization. Moreover, comparing with the *Lq* (1/2 < *q* < 1) penalties, the harmonic regularization has the property that its first derivative is finite at origin, which implies that the corresponding regularization problem can be efficiently solved via the direct seeking techniques.

## 3. The Harmonic Regularization Algorithm for the Cox Model

In this section, we propose a generalized path seeking algorithm of the harmonic regularization for Cox's model. As mentioned in the last section, when *Lq* (0 < *q* < 1) regularization is to be applied, an inevitable difficulty is how to efficiently solve the nonconvex optimization problem caused by the *Lq* (0 < *q* < 1) regularization (It is easy to see that in the case of *Lq* (*q* > 1) regularization is applied, the penalty term becomes convex). Fortunately, direct path seeking makes it possible to overcome that difficulty. Direct path seeking, which sequentially constructs a path directly in the parameter space, closely approximates that for a penalty function without having to repeatedly solve numerical optimization problems. Popular path seeking based on squared-error includes partial least squares regression (PLS, [23]), forward stepwise regression [22], least angle regression [25], piecewise linear path [14], and gradient boosting. Friedman [26] proposed the generalized path seeking, which can produce solutions that closely approximate those for any convex loss function and nonconvex constraints. The advantages of path seeking methods provide us a new way to solve the problem of regularization with nonconvex penalty. We will propose a new generalized path seeking method to solve the harmonic regularization.

We let

(7) $$\begin{array}{c} P\left(\beta \right)=\overset{p}{\underset{j=1}{\sum }}P_{j}\left(\left|\beta_{j}\right|\right), \end{array}$$

where $P_{j}(\beta_{j})=\sqrt{\left(2/a\left(a-1\right)\right)\left|\beta_{j}\right|+{\left(\left(2-a\right)/\left(a-1\right)\right)}^{2}}-\left(2-a\right)/\left(a-1\right)$. Note that

(8) $$\begin{array}{c} \frac{\partial P_{j}\left(\beta_{j}\right)}{\partial \left|\beta_{j}\right|}=\frac{1}{\sqrt{2a\left(a-1\right)\left|\beta_{j}\left(v\right)\right|+a^{2}{\left(a-2\right)}^{2}}}>0, \end{array}$$

which shows that each additive term *P*_*j*_(*β*_*j*_) is a monotonically increasing function of absolute value of its argument. This implies that the net regularization penalty function we have suggested meets the validity of the general path seeking algorithm [11]. Let *v* measure length along the path and Δ*v* > 0 a small increment. Define

(9) gj(v)=−∂Rβ∂βjβ=βv=1n∑i=1n(y^i−x^iβ)x^ij,pjv=∂Pj(βj)∂|βj|β=β(v)=12aa−1βjv+a2a−22,

and let

(10) $$\begin{array}{c} \tau_{j}\left(v\right)=\frac{g_{j}\left(v\right)}{p_{j}\left(v\right)}. \end{array}$$

Then, we give the harmonic regularization algorithm procedures for the Cox model as follows:

1. initialize *v* = 0, *k* = 0, {*β*_*j*_^*k*^(*v* = 0) = 0}_*j*=1_^*p*^;
2. compute $\hat{x}$ and $\hat{y}$ based on (3) using the current value *β*_*j*_^*k*^(*v*), *j* = 1,…, *p*;
3. loop {
4.  compute *τ*_*j*_(*v*) = *g*_*j*_(*v*)/*p*_*j*_(*v*), *j* = 1,…, *p*;
5.  
*S* = {*j*∣*τ*_*j*_(*v*) × *β*_*j*_^*k*^(*v*) < 0, *j* = 1,…, *p*};
6.  if (*S* = empty)  *j* ^*^ = arg max⁡_*j*_⁡|*τ*_*j*_(*v*)|;
7.  else *j* ^*^ = arg max⁡_*j*∈*S*_⁡|*τ*_*j*_(*v*)|;
8.  
*β*_j^*^_^k^(*v* + Δ*v*) = *β*_j^*^_^k^(*v*) + Δ*v* × sign⁡(*τ*_j^*^_(*v*));
9.  
*β*_*j*_^*k*^(*v* + Δ*v*) = *β*_*j*_^*k*^(*v*), *j* = 1,…, *n* and *j* ≠ *j* ^*^;
10.  
*v* ← *v* + Δ*v*;
11. } until *τ*_*j*_(*v*) = 0, *j* = 1,…, *p*;
12. *k* ← *k* + 1, and go back to Step 2 until the convergence criterion is met.

In the above algorithm, after Step 2, at each step those coefficients *β*_*j*_^*k*^(*v*) with sign opposite to that of the corresponding *τ*_*j*_(*v*) are identified. When the set *S* is empty, the coefficient corresponding to the largest component of {*τ*_*j*_(*v*)}_*j*=1_^*n*^, an absolute value is selected at Step 6. And when there are one or more elements in the set *S*, the coefficient with corresponding largest *τ*_*j*_(*v*) within this subset is instead selected. The selected coefficient is the increments by a small amount in the direction of the sign of its corresponding *τ*_*j*∗_(*v*) while all other coefficients remain unchanged, yielding the solution for the next path point *v* + Δ*v*. The iterations continue until all components of *τ*_*j*_(*v*) = 0 and the algorithm then reaches a regularized solution for the harmonic regularized Cox model.

## 4. Simulation

### 4.1. Selection of the Shrinkage Parameter *a* and the Tuning Parameter *λ*

To select the shrinkage parameter a and the tuning parameter *λ*, we use the maximization of the cross-validation partial likelihood (CVPL) method proposed by van Houwelingen et al. [27], which is defined as

(11) $$\begin{array}{c} \text{CVPL}\left(a,\lambda \right)=-\frac{1}{k}\overset{k}{\underset{i=1}{\sum }}\left\{l\left(\beta_{\left(-i\right)}\left(a,\lambda \right)\right)-l_{\left(-i\right)}\left(\beta_{\left(-i\right)}\left(a,\lambda \right)\right)\right\}, \end{array}$$

where *β*_(−*i*)_(*a*, *λ*) represents the estimation of *β* based on the harmonic regularization procedure with the parameters *a* and *λ* from the data without the *i*th subject. The terms *l*(*β*) and *l*_(−*i*)_(*β*) are the log partial likelihoods with all the subjects and without the *i*th subject, respectively. The optimal value of the parameters *a* and *λ* are chosen to maximize the sum of the contributions of each subject to the log partial likelihood over a grid of (*a*, *λ*). CVPL is the special case of a more general cross-validated likelihood approach for model selection and has been demonstrated to perform well in prediction in the context of the penalized Cox regression.

### 4.2. Model Validation Measures

The performance measures of censored survival data is more complicated: the measure can only be computed if the case is not right censoring. Thus, several specially designed measure method have been proposed in the literatures. In this paper, we employ the integrated brier score (IBS) [28] and the concordance index (CI) [29] to evaluate the prediction ability of the regularization methods.

*Integrated Brier Score (IBS).* The brier score (BS) is defined as a function of time *t* > 0 by

(12) BSt=1n∑i=1nS^t ∣ Xi21(ti≤t∧δi=1)G^(ti)1−S^t ∣ Xi21(ti>t)G^(t)ddddddd+1−S^t ∣ Xi21ti>tG^t,

where $\hat{G}(\cdot )$ denotes the Kaplan-Meier estimation of the censoring distribution and $\hat{S}\left(\cdot |X_{i}\right)$ stands to estimate survival for patient *i*. Note that the BS(*t*) is dependent on the point in time *t*, and its values are between 0 and 1. Good predictions at time *t* result in small values of BS. The integrated brier score (IBS) is given by

(13) $$\begin{array}{c} \text{IBS}=\frac{1}{\max\left(t_{i}\right)}\overset{\max\left(t_{i}\right)}{\underset{0}{\int }}\text{BS}\left(t\right)dt. \end{array}$$

The IBS is used to assess the goodness of the predicted survival functions of all observations at every time between 0 and max⁡⁡(*t*_*i*_). 

*Concordance Index (CI).* The concordance index (CI) can be interpreted as the fraction of all pairs of subjects which predicted survival times are correctly ordered among all subjects that can actually be ordered. By the CI definition, we can determine *t*_*i*_ > *t*_*j*_ when *f*_*i*_ > *f*_*j*_ and *δ*_*j*_ = 1, where *f*(·) is survival function. The pairs for which neither *t*_*i*_ > *t*_*j*_ nor *t*_*i*_ < *t*_*j*_ can be determined are excluded from the calculation of CI. Thus, the CI is defined as

(14) $$\begin{array}{c} \text{CI}=\frac{\sum_{i}^{}\;\sum_{j}^{}1\left(f_{i}<f_{j}\wedge \delta_{i}=1\right)}{\sum_{i}^{}\;\sum_{j}^{}1\left(t_{i}<t_{j}\wedge \delta_{i}=1\right)}. \end{array}$$

Note that the values of CI are between 0 and 1, the perfect predictions of the building model would lead to 1 while have the CI value of 0.5 at random.

### 4.3. Analyses of the Simulated Data

In this section, we evaluate the performance of the harmonic regularization method for the Cox model in simulation study. We generate high-dimensional and low sample size data which contain many irrelevant features. Six methods are compared with our proposed harmonic regularization approach (HRA): the Lasso penalty (*L*_1_), the smoothly clipped absolute deviation penalty (SCAD), the minimax concave penalty (MCP), the adaptive Lasso (A-Lasso), the elastic net (*L*_en_), and the *L*_1/2_ penalty (*L*_1/2_).

We adopted the Cox model simulation scheme in Bender's work [30]. The data generation procedure is as follows.

**Step 1 .**

We generated the vectors *γ*_*i*0_, *γ*_*i*1_,…, *γ*_*ip*_  (*i* = 1,…, *n*) independently from a standard normal distribution and the predictor vector *x*_*i*_ is generated by $x_{ij}=\gamma_{ij}\sqrt{1-\rho }+\gamma_{i0}\sqrt{\rho }$  (*j* = 1,…, *p*), where *ρ* is the correlation parameter of the predictor vectors.

**Step 2 .**

The survival time *t*_*i*_′  (*i* = 1,…, *n*, *n* indicates the sample size) is constructed from a uniformly distributed variable *U* by *t*_*i*_′ = (1/*γ*)log⁡(1 − (*γ* × log⁡(*U*))/(*ω*exp⁡(*x*_*i*_ *β* + *σ* × *ε*))), where *γ* is the shape parameter, *ω* is the scale parameter, *β* is the ground-true regression coefficients, *ε* is the independent random error generated from *N*(0,1), and *σ* is the parameter which controls the signal to noise.

**Step 3 .**

Censoring time point *t*_*i*_′′  (*i* = 1,…, *n*) is obtained from an exponential distribution *E*(*θ*), where *θ* is determined by the specify censoring rate.

**Step 4 .**

Here we define *t*_*i*_ = min⁡(*t*_*i*_′, *t*_*i*_′′) and *δ*_*i*_ = *I*(*t*_*i*_′ ≤ *t*_*i*_′′), the observed data represented as (*t*_*i*_, *δ*_*i*_, *x*_*i*_) for the Cox model (1) are generated.

In every simulation, the dimension *p* of the predictor vector *x*_*i*_ is 1000, and the first five true coefficients are nonzero: *β*_1_ = 1, *β*_2_ = 0.8, *β*_3_ = −1, *β*_4_ = −0.8, *β*_5_ = 1, and *β*_*j*_ = 0  (6 ≤ *j* ≤ 1000). About 25% of the data are right censored. We consider the cases with the training sample sizes *n* = 100, 150, 200, the correlation coefficient *ρ* = 0.1, 0.5, and the noise control parameter *σ* = 0.2, 0.5, respectively. To assess the variability of the experiment, each method is evaluated on a test set including 100 random generated samples.

The estimation of the optimal tuning parameter *λ* in the regularization models can be done in many ways and is often done by *k*-fold cross-validation (CV). Note that the choice of *k* will depend on the size of the training set. In our experiments, we use 10-fold cross-validation (*k* = 10). The elastic net method has two tuning parameters; we need to cross-validate on a two-dimensional surface.

Table 1 shows the average number of variable selected and the recovery rate by each regularization method in 500 runs. The recovery rate is defined as the ratio of the average number of the selected relevant variables (*x*_1_–*x*_5_) to the average number of the selected variables [9]. As shown in Table 1, when the sample size *n* increases, the prediction performances of all the seven methods are improved. For example when *ρ* = 0.1 and *σ* = 0.2, the average of the variables selected by the harmonic regularization method decreased from 6.6 to 5.8 and its recovery rate is improved from 0.72 to 0.86 with the sample sizes *n* increased from 100 to 200. When the correlation parameter *ρ* and the noise parameter *σ* increase, the variable selection performances of all the seven methods are decreased. For example, when *ρ* = 0.1 and *N* = 200, the average of the recovery rate from the harmonic method decreased from 0.86 to 0.71, in which *σ* increased from 0.2 to 0.5. When *σ* = 0.5 and *n* = 150, the average of the recovery rate of the harmonic method decrease from 0.63 to 0.50, in which *ρ* increased from 0.1 to 0.5. Moreover, in our simulation, the influence of the noise may be slightly larger than that of the variable correlation for the prediction performance of all the seven methods. On the other hand, at the same parameter setting case, the recovery rates of the harmonic method and *L*_1/2_ penalty are almost better than the results of the other five methods. For example, when *ρ* = 0.1, *σ* = 0.2 and *n* = 100, the recovery rate of the harmonic method is 0.72 much better than 0.14, 0.43, 0.54, 0.28, and 0.13 got by the Lasso, SCAD, MCP, adaptive Lasso, and elastic net, respectively, slight better than 0.71 got by *L*_1/2_ penalty method.

To evaluate prediction performance of the seven regularization methods for the Cox model, we presented their average IBC and CI values on the simulated datasets among 500 times in Table 2.

In terms of IBC and CI, for different parameters' settings, no methods almost performed better than others, but their prediction performances are only small differences. For example, when *ρ* = 0.1, *σ* = 0.2, and *n* = 150, the average of IBS from the harmonic method is 0.079, better than 0.081, 0.087, 0.084, 0.08, 0.08, and 0.084 got by Lasso, SCAD, MCP, adaptive Lasso, and elastic net and *L*_1/2_ penalty. When *ρ* = 0.1, *σ* = 0.2, and *n* = 100, the average of CI from the harmonic method is 0.845, better than 0.749, 0.788, 0.822, 0.757, and 0.838 got by Lasso, SCAD, MCP, adaptive Lasso, and *L*_1/2_, but slight worse than 0.851 got by elastic net penalty method.

Combined with the results reported in Table 1, we concluded that the harmonic penalized method showed better or equivalent predictive performance than the other regularization methods.

### 4.4. Analysis of the Real Microarray Datasets

In this section, we evaluated the performance of the harmonic regularization methods on the real survival gene expression datasets. Four publicly available datasets are used in this part. A brief description of these datasets is given below and summarized in Table 3.

*Diffuse Large B-cell Lymphoma Dataset (DLBCL) 2002*. This dataset published by Rosenwald et al. [31]. The dataset consists of 240 samples from patients. For each sample, 7399 gene expression measurements were obtained. The clinical outcome was survival time, either observed or censored.

*Diffuse Large B-cell Lymphoma Dataset (DLBCL) 2003*. This dataset is from Rosenwald et al. [32]. It consists of 92 lymphoma patients, and each patient has 8810 genes.

*Lung Cancer Dataset.* The lung cancer dataset is from Beer et al. [33]. It consists of gene expressions of 4966 genes for 83 patients. The survival time as well as the censoring status is available.

*AML Dataset.* The AML dataset is from Bullinger et al. [34]. It contains the expression profiles of 6283 genes for 116 patients, and the number of censored cases is 49.

We evaluated the prediction accuracies of the seven estimated regularization methods using random partition: a training set of about 2/3 of the patients used for estimation and a test set of about 1/3 of the patients used for testing of the prediction capability. For estimating *λ*, we employed the five-fold cross-validation scheme using the training set. We repeated each procedure 200 times.

Table 4 reports the average number of genes selected by each method. The harmonic regularization method performs better than those of *L*_1_ type methods (Lasso, SCAD, MCP, adaptive Lasso, and elitist net), and slightly better than that of *L*_1/2_ penalty. As shown in Table 4, for DLBCL (2002) dataset, the harmonic penalized methods selected about 71 genes, compared to about 174, 129, 129, 146, and 180 about five Lasso, SCAD, MCP, adaptive Lasso and elitist net, slightly better than 76 got by *L*_1/2_ penalty. For DLBCL (2003) and AML datasets, the best one is *L*_1/2_ penalty and the second is harmonic methods.

To assess predictive performance, we summarize the results of IBS and CI obtained by the seven methods, respectively, in Table 5. Both the results of IBS and CI, the results of all regularization methods, were not much different and the elitist net and harmonic penalized method almost outperforms than other five penalized methods. Combined with the results reported in Tables 4 and 5, we concluded that the harmonic penalized method selected the smaller subset of the key genes while give best or equivalent predictive performance.

## 5. Conclusion

Variable selection is a fundamental problem in statistics and machine learning, and the regularization method is one of the ways to solve this problem. Generally speaking, a regularization algorithm is always a combination of a loss function and a penalty function in the past research and applications. Particularly, in the procedure of variable selection, the harmonic regularization is like a net which can always catch the correct model. This demonstrates the stronger sparsity and better correctness of the harmonic regularization. We have provided a serous of simulations to demonstrate that *L*_1_ type regularization methods are inefficient; the harmonic regularization and *L*_1/2_ penalty methods proved are efficient and effective.

In the simulation part, we use four real datasets. There are the DLBCL (2002), the DLBCL (2003), the Lung cancer, and the AML. Results indicate that our harmonic regularization algorithm is very competitive in analyzing high dimensional survival data in terms of sparsity. Simulation results indicate that the harmonic penalized Cox model is very competitive in analyzing high dimensional survival data, because it was able to reduce the size of the predictor even further at moderate costs for the prediction accuracy [8]. The harmonic penalized Cox model will provide an efficient tool in building a prediction model for survival time based on high dimensional biological data.

## Figures and Tables

**Table 1:** Average number of the variable selected and the recovery rate by the seven regularization methods on the simulated data in 500 runs.

Corr.	Size	Average of variable selected						
		*L*_1_	SCAD	MCP	A-Lasso	Len	*L*_1/2_	HRA
ρ = 0.1 σ = 0.2	100	33.4	11.6	9.2	17.8	35.8	**6.6**	**6.6**
	150	29.7	9.8	7.9	12.8	31.2	**5.9**	6.3
	200	24.4	8.4	6.1	9.4	24.8	**5.7**	5.8

ρ = 0.1 σ = 0.5	100	43.2	14.8	11.7	22.9	46.9	9.9	**9.7**
	150	34.5	11.2	8.7	16.5	36.3	**7.2**	7.3
	200	26.7	9.7	7.7	11.4	28.2	**6.5**	7

ρ = 0.5 σ = 0.2	100	45.1	15.1	12.2	27.2	47.8	**10.8**	10.9
	150	39.3	11.9	10.8	20.1	44.3	8.4	**8.3**
	200	27.1	10.1	8.4	12.6	30.6	**7.3**	7.7

ρ = 0.5 σ = 0.5	100	55.6	17.7	16	32.7	56.3	**13.9**	15.2
	150	47.3	15.9	14.8	25.9	48.6	**9.4**	10
	200	36.8	13.7	12.5	19.5	41.6	**7.8**	**7.8**

Corr.	Size	Recovery rate						
		*L*_1_	SCAD	MCP	A-Lasso	Len	*L*_1/2_	HRA

ρ = 0.1 σ = 0.2	100	0.14	0.43	0.54	0.28	0.13	0.71	**0.72**
	150	0.16	0.51	0.63	0.39	0.16	**0.8**	0.79
	200	0.2	0.59	0.81	0.53	0.2	**0.87**	0.86

ρ = 0.1 σ = 0.5	100	0.11	0.33	0.42	0.21	0.1	0.5	**0.51**
	150	0.14	0.44	0.57	0.3	0.13	**0.65**	0.63
	200	0.18	0.51	0.64	0.43	0.17	0.7	**0.71**

ρ = 0.5 σ = 0.2	100	0.11	0.33	0.4	0.18	0.1	**0.46**	0.45
	150	0.12	0.42	0.46	0.24	0.11	0.59	**0.6**
	200	0.18	0.49	0.59	0.39	0.16	0.62	**0.63**

ρ = 0.5 σ = 0.5	100	0.08	0.28	0.31	0.15	0.08	**0.35**	0.32
	150	0.1	0.31	0.33	0.19	0.1	**0.51**	0.5
	200	0.13	0.36	0.4	0.25	0.12	**0.61**	**0.61**

**Table 2:** Average IBS and CI results of by the seven regularization methods on the simulated data in 500 runs.

Corr.	Size	Average IBS						
		*L*_1_	SCAD	MCP	A-Lasso	Len	*L*_1/2_	HRA
ρ = 0.1 σ = 0.2	100	0.084	0.094	0.086	0.084	**0.082**	0.091	0.086
	150	0.081	0.087	0.084	0.08	0.08	0.084	**0.079**
	200	0.078	0.086	0.079	0.083	**0.076**	0.078	**0.076**

ρ = 0.1 σ = 0.5	100	0.096	**0.092**	0.097	0.096	0.094	0.098	0.093
	150	0.092	0.091	0.094	0.094	**0.087**	0.089	0.09
	200	0.088	0.088	0.086	0.087	**0.085**	0.086	0.086

ρ = 0.5 σ = 0.2	100	0.105	0.098	0.101	0.102	0.097	0.101	**0.094**
	150	0.098	0.096	0.099	0.102	**0.092**	0.098	0.091
	200	0.091	0.092	0.096	0.096	**0.089**	0.095	0.09

ρ = 0.5 σ = 0.5	100	0.108	0.103	0.108	0.106	0.099	0.01	**0.098**
	150	0.101	0.097	0.1	0.096	**0.093**	0.094	0.097
	200	0.084	0.094	0.086	0.084	**0.082**	0.091	0.086

Corr.	Size	Average CI						
		*L*_1_	SCAD	MCP	A-Lasso	Len	*L*_1/2_	HRA

ρ = 0.1 σ = 0.2	100	0.749	0.788	0.822	0.757	**0.851**	0.838	0.845
	150	0.832	0.853	0.869	0.838	0.868	0.865	**0.87**
	200	0.85	0.847	0.857	0.859	**0.864**	0.859	0.862

ρ = 0.1 σ = 0.5	100	0.728	0.758	**0.767**	0.716	0.727	0.761	0.763
	150	0.82	0.841	0.833	0.831	**0.853**	0.847	0.837
	200	0.847	0.857	0.862	0.846	**0.869**	0.862	0.866

ρ = 0.5 σ = 0.2	100	0.726	**0.758**	0.752	0.745	0.752	**0.758**	0.748
	150	0.781	0.818	0.821	0.793	0.819	0.813	**0.821**
	200	0.786	0.835	0.826	0.792	**0.839**	0.824	0.828

ρ = 0.5 σ = 0.5	100	0.699	0.712	0.701	0.685	**0.719**	0.716	0.714
	150	0.766	0.777	0.817	0.788	0.814	**0.818**	0.814
	200	0.776	0.801	0.82	0.808	**0.821**	0.819	0.819

**Table 3:** The gene expression datasets are used in experiments.

Datasest	Number of genes	Number of samples	Number of censored
DLBCL (2002)	7399	240	102
DLBCL (2003)	8810	92	28
Lung cancer	7129	86	62
AML	6283	116	49

**Table 4:** Results of the gene selected by the seven methods on the four public datasets.

Datasest	*L*_1_	SCAD	MCP	A-Lasso	Len	*L*_1/2_	HRA
DLBCL (2002)	174	129	129	146	180	76	**71**
DLBCL (2003)	138	106	95	168	142	**32**	37
Lung cancer	188	104	97	233	196	56	**48**
AML	161	120	110	176	166	**65**	70

In bold is the best performance.

**Table 5:** The IBS results obtained by the seven methods on the four public datasets.

Datasets	Average IBS						
	*L*_1_	SCAD	MCP	A-Lasso	Len	*L*_1/2_	HRA
DLBCL (2002)	0.207	0.205	0.205	0.205	**0.198**	0.203	0.205
DLBCL (2003)	0.121	0.119	0.12	0.12	0.12	**0.118**	0.119
Lung cancer	0.169	0.161	0.167	0.164	**0.169**	0.163	0.161
AML	0.174	0.174	0.173	0.172	**0.171**	0.173	**0.171**

Datasets	Average CI						
	*L*_1_	SCAD	MCP	A-Lasso	Len	*L*_1/2_	HRA

DLBCL (2002)	0.553	0.554	0.564	0.555	**0.568**	0.563	0.566
DLBCL (2003)	0.583	0.604	0.586	0.589	**0.606**	0.603	**0.606**
Lung cancer	0.628	0.634	0.666	0.646	**0.675**	0.673	0.674
AML	0.599	0.611	0.634	0.626	0.641	0.638	**0.643**

In bold-the best performance.

## References

[1] Leung E. L., Cao Z. W., Jiang Z. H., Zhou H., Liu L. (2013). Network-based drug discovery by integrating systems biology and computational technologies. *Briefings in Bioinformatics*.

[2] Cox D. R. (1972). Regression models and life-tables. *Journal of the Royal Statistical Society B. Methodological*.

[3] Cox D. R. (1975). Partial likelihood. *Biometrika*.

[4] Fan J., Li R. (2001). Variable selection via nonconcave penalized likelihood and its oracle properties. *Journal of the American Statistical Association*.

[5] Tibshirani R. (1996). Regression shrinkage and selection via the lasso. *Journal of the Royal Statistical Society B. Methodological*.

[6] Zhang H. H., Lu W. (2007). Adaptive Lasso for Cox's proportional hazards model. *Biometrika*.

[7] Zhang C. H. (2010). Nearly unbiased variable selection under minimax concave penalty. *The Annals of Statistics*.

[8] Liu Z., Jiang F., Tian G., Wang S., Sato F., Meltzer S. J., Tan M. (2007). Sparse logistic regression with Lp penalty for biomarker identification. *Statistical Applications in Genetics and Molecular Biology*.

[9] Xu Z. B., Zhang H., Wang Y., Chang X. Y., Liang Y. (2010). L 1/2 regularization. *Science in China Series F: Information Sciences*.

[10] Xu Z. B., Chang X. Y., Xu F. M. (2012). L-1/2 regularization: a thresholding representation theory and a fast solver. *IEEE Transaction on Neural Networks and Learning Systems*.

[11] Tibshirani R. (1997). The lasso method for variable selection in the Cox model. *Statistics in Medicine*.

[12] Gui J., Li H. (2005). Penalized Cox regression analysis in the high-dimensional and low-sample size settings, with applications to microarray gene expression data. *Bioinformatics*.

[13] Zou H., Hastie T. (2005). Regularization and variable selection via the elastic net. *Journal of the Royal Statistical Society B: Statistical Methodology*.

[14] Rosset S., Zhu J. (2007). Piecewise linear regularized solution paths. *Annals of Statistics*.

[15] Zhang T. (2010). Analysis of multi-stage convex relaxation for sparse regularization. *Journal of Machine Learning Research (JMLR)*.

[16] Mazumder R., Friedman J. H., Hastie T. (2011). SparseNet : coordinate descent with non-convex penalties. *Journal of the American Statistical Association*.

[17] Liang Y., Liu C., Luan X.-Z., Leung K.-S., Chan T.-M., Xu Z.-B., Zhang H. (2013). Sparse logistic regression with a L1/2 penalty for gene selection in cancer classification. *BMC Bioinformatics*.

[18] Liu C., Liang Y., Luan X. Z., Leung K. S., Chan T. M., Xu Z. B., Zhang H. (2013). The L1/2 regularization method for variable selection in the Cox model. *Applied Soft Computing*.

[19] Fu W. J. (1998). Penalized regressions: the bridge versus the lasso. *Journal of Computational and Graphical Statistics*.

[20] Blumensath T., Yaghoobi M., Davies M. E. Iterative hard thresholding and L_0_ regularisation.

[21] Sohn I., Kim J., Jung S.-H., Park C. (2009). Gradient lasso for Cox proportional hazards model. *Bioinformatics*.

[22] Zhao P., Yu B. (2007). Stagewise lasso. *Journal of Machine Learning Research*.

[23] Donoho D. L., Huo X. (2001). Uncertainty principles and ideal atomic decomposition. *IEEE Transactions on Information Theory*.

[24] Donoho D. L., Elad E. (2003). Maximal sparsity representation via L1 minimization. *Proceedings of the National Academy of Sciences of the United States of America*.

[25] Efron B., Hastie T., Johnstone I., Tibshirani R. (2004). Least angle regression. *The Annals of Statistics*.

[26] Friedman J. H. (2008). Fast sparse regression and classification.

[27] van Houwelingen H. C., Bruinsma T., Hart A. A. M., van't Veer L. J., Wessels L. (2006). Cross-validated Cox regression on microarray gene expression data. *Statistics in Medicine*.

[28] Graf E., Schmoor C., Sauerbrei W., Schumacher M. (1999). Assessment and comparison of prognostic classification schemes for survival data. *Statistics in Medicine*.

[29] Harrel F. E. (2001). *Regression Modeling Strategies, with Applications to Linear Models, Logistic Regression, and Survival Analysis*.

[30] Bender R., Augustin T., Blettner M. (2005). Generating survival times to simulate Cox proportional hazards models. *Statistics in Medicine*.

[31] Rosenwald A., Wright G., Chan W. C. (2002). The use of molecular profiling to predict survival after chemotherapy for diffuse large B-cell lymphoma. *The New England Journal of Medicine*.

[32] Rosenwald A., Wright G., Wiestner A., Chan W. C., Connors J. M., Campo E., Gascoyne R. D., Grogan T. M., Muller-Hermelink H. K., Smeland E. B., Chiorazzi M., Giltnane J. M., Hurt E. M., Zhao H., Averett L., Henrickson S., Yang L., Powell J., Wilson W. H., Jaffe E. S., Simon R., Klausner R. D., Montserrat E., Bosch F., Greiner T. C., Weisenburger D. D., Sanger W. G., Dave B. J., Lynch J. C., Vose J., Armitage J. O., Fisher R. I., Miller T. P., LeBlanc M., Ott G., Kvaloy S., Holte H., Delabie J., Staudt L. M. (2003). The proliferation gene expression signature is a quantitative integrator of oncogenic events that predicts survival in mantle cell lymphoma. *Cancer Cell*.

[33] Beer D. G., Kardia S. L. R., Huang C.-C., Giordano T. J., Levin A. M., Misek D. E., Lin L., Chen G., Gharib T. G., Thomas D. G., Lizyness M. L., Kuick R., Hayasaka S., Taylor J. M. G., Iannettoni M. D., Orringer M. B., Hanash S. (2002). Gene-expression profiles predict survival of patients with lung adenocarcinoma. *Nature Medicine*.

[34] Bullinger L., Döhner K., Bair E., Fröhling S., Schlenk R. F., Tibshirani R., Döhner H., Pollack J. R. (2004). Use of gene-expression profiling to identify prognostic subclasses in adult acute myeloid leukemia. *The New England Journal of Medicine*.

